# Optimization of In Vitro White Spot Lesion Protocol: Comparative Diagnostics via Visual Inspection, Scanning Electron Microscopy, and Fluorescence Methods

**DOI:** 10.3390/diagnostics16121795

**Published:** 2026-06-10

**Authors:** Karrar K. Alseedi, Noor M. H. Garma

**Affiliations:** Department of Orthodontics, College of Dentistry, University of Baghdad, Baghdad 10071, Iraq; karrar.yasir2403p@codental.uobaghdad.edu.iq

**Keywords:** white spot lesions, hydroxyethylcellulose, DIAGNOdent, ICDAS II, scanning electron microscope, enamel demineralization

## Abstract

**Background/Objectives:** The creation of clinically relevant in vitro white spot lesions (WSLs) is essential for the reliable evaluation of diagnostic devices and remineralization therapies. This study aims to optimize the in vitro WSL creation protocol across various demineralization periods to produce lesions comparable to post-orthodontic WSLs, using different diagnostic methods. **Methods**: Forty extracted upper first premolars were randomly allocated into four groups (ten per group) and exposed to hydroxyethylcellulose (HEC) gel for demineralization periods of 7, 10, 14, and 21 days. Lesions were evaluated by ICDAS II, DIAGNOdent pen, and a Scanning Electron Microscope (SEM). The correlations among the diagnostic methods were analyzed. **Results:** According to ICDAS II scoring, a score of 1 was predominant in the 7-day group, while the others exhibited a score of 2. DIAGNOdent readings increased significantly with longer durations (G7 mean: 7.80 ± 2.04; G21 mean: 82.70 ± 13.11). SEM analysis indicated an increase in lesion depth with extended exposure durations (G7 mean: 46.59 ± 2.72; G21 mean: 101.7 ± 9.75). Nevertheless, the rate of increase in lesion depth diminished with prolonged exposure. A strong correlation was observed between lesion depth and DIAGNOdent readings (r = 0.90). Additionally, ICDAS II demonstrated moderate correlations with DIAGNOdent and lesion depth (r = 0.69 and 0.66, respectively). **Conclusions:** A 10-day demineralization period was identified as the most favorable and reproducible, producing lesions with an intact surface and consistent with DIAGNOdent criteria. This duration may serve as the optimal model for evaluating intensive remineralization therapies and diagnostic methods prior to clinical implementation.

## 1. Introduction

White spot lesions (WSLs) are the earliest visible sign of enamel demineralization and are frequently observed in patients undergoing orthodontic treatment, with prevalence increasing with treatment duration [1,2]. They often appear as opaque, chalky areas in plaque-retentive regions of the tooth surface, posing both clinical and esthetic challenges. WSLs are characterized by a radiolucent, porous subsurface enamel, whereas the outer enamel remains intact and radiopaque [3]. In laboratory studies, artificial WSLs can be created using various demineralization models, including biofilm models, chemical models (acetate buffer solution or acidified gel), and pH cycling models [3,4,5,6,7,8,9]. Hydroxyethylcellulose (HEC) gel combined with lactic acid (acidified gel) is a widely used method to produce subsurface artificial WSLs in numerous studies due to its simplicity and reproducibility [5,9,10,11]. The depth and extent of mineral loss in WSLs are significantly influenced by the duration of acid exposure and temperature; longer exposure times and higher temperatures result in deeper lesions and greater mineral loss. However, increased surface porosity and loss of subsurface WSL characteristics may occur [12,13]. Increased surface porosity can eventually turn an intact surface WSL into an eroded or cavitated lesion, reducing hardness and mechanical strength, thereby diminishing the effectiveness of non-invasive treatment and necessitating a restorative option [14,15]. Therefore, the time-dependent pattern is critical for simulating early carious lesions and testing the efficacy of diagnostic tools and remineralizing therapies.

WSLs can be assessed using various diagnostic approaches, among which is the International Caries Detection and Assessment System (ICDAS II). ICDAS II is a commonly used visual scoring system for dental caries, including smooth-surface caries, in both in vivo and in vitro studies. The scoring system range (0–6) is classified according to the lesion severity reflected by enamel opacity and breakdown. However, examiner subjectivity and viewing conditions may influence its outcomes [1]. Therefore, it is often validated by histological sectioning, micro-computed tomography, and other diagnostic methods [2]. Micro-computed tomography (Micro-CT) is a non-destructive method that allows for repeated scans without damaging the sample, which is helpful in longitudinal remineralization studies. However, it may be affected by beam-hardening artifacts unless appropriate filters and corrections are applied. On the other hand, Transverse Micrography (TMR) is considered the gold standard for mineral content. However, sample destruction to thin sections is required to quantify mineral content [3]. Moreover, the limited availability and cost of both Micro-CT and TMR may limit widespread use.

In addition, a Scanning Electron Microscope (SEM) is another diagnostic method that is used to measure the depth and surface morphology of these artificial lesions, producing a high-resolution image that shows the surface and subsurface change in response to the demineralization periods [4]. However, it is a destructive method, has limited ability to measure mineral content of the entire lesion, and is unsuitable for clinical application. Alternatively, non-invasive techniques, including Laser Fluorescence (LF) detecting devices (e.g., DIAGNOdent), have been used as an additional diagnostic method to monitor the dynamics of WSL de- and remineralization and the reading change with the severity of the lesion [5,6]. However, their reading values may be compromised by plaque, staining, or an orthodontic appliance, leading to variable sensitivity [7].

To evaluate the effectiveness of present and newly developed remineralizing therapies, the in vitro WSLs should be clinically relevant. According to the DIAGNOdent (DD) guidelines, most of the post-orthodontic WSLs with DIAGNOdent scores of less than 13 and a ICDAS II score of 1 undergo spontaneous remineralization with normal prophylaxis [8,9], whereas the 13–24 DD score requires intensive prophylaxis, such as varnish fluoride application. On the other hand, scores of more than 25 DD need more aggressive treatment, like ICON, micro-abrasion, conservative filling, or others [10]. Consequently, the limitations of the various diagnostic methods have increased the demand for well-designed studies that enhance WSLs evaluation, correlate the findings of different techniques, and improve the reliability and clinical relevance of in vitro WSLs [1,7,11].

This study aimed: (1) to develop in vitro WSLs with an intact outer layer, the deepest subsurface decay, an ICDAS II score 2, and a 13–24 DD score range comparable to clinical post-orthodontic WSLs, which require intensive therapeutic measures via a hydroxyethylcellulose acidified gel at various interval protocols; (2) to evaluate the correlation among the ICDAS scores, DIAGNOdent pen scores, and SEM observations of WSLs depth developed at different intervals.

Accordingly, the null hypotheses were as follows: (1) there is no significant difference in the surface and subsurface characteristics among the selected durations; (2) there is no significant difference in the WSLs depth among the selected durations; (3) there is no significant correlation among the ICDAS scores, DIAGNOdent fluorescence values, and SEM-measured WSL depth.

## 2. Materials and Methods

### 2.1. Sample Size Justification

The sample size was 40 teeth (10 teeth per group). It was selected based on a sensitivity analysis using G*Power v3.1.9.7 (Franz Faul, Uni Kiel, Kiel, Germany), with a one-way ANOVA at an alpha level of 0.05 and 80% power to detect a large effect size (f ≈ 0.55).

### 2.2. Sample Preparation

**Figure 1:**
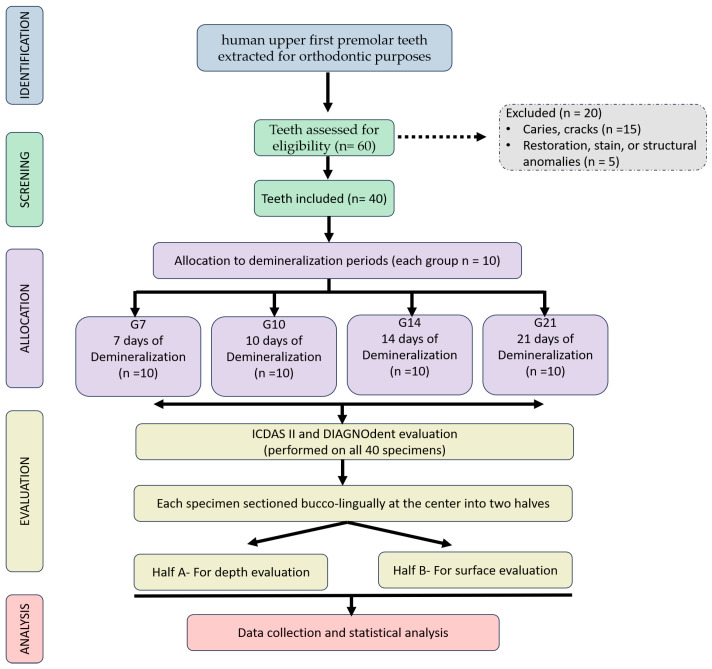
Flow diagram of the experimental design.

Following the receipt of ethical approval from the Research Ethics Committee of the College of Dentistry, University of Baghdad, Iraq (Ref. number: 1030; approved on 25 February 2025), human upper first premolar teeth extracted for orthodontic purposes were collected from Orthodontic Department, College of Dentistry, University of Baghdad, and from a private orthodontic clinic in Wasit, Iraq, with an age range of 15 to 30 years. Post-extraction, the teeth were meticulously rinsed to remove any tissue and blood residues. Subsequently, the teeth were immersed in 1% Chloramine-T trihydrate for seven days and then stored in ultra-pure water in accordance with ISO/11405:2015 standards [16]. All teeth underwent examination using a stereomicroscope at ×10 magnification. The criteria for tooth selection included an intact buccal surface for WSL development and an intact lingual surface for DIAGNOdent pen calibration, with no prior chemical treatment, absence of visible caries, and no enamel cracks or irregularities (Figure 1).

### 2.3. White Spot Lesion Creation

Initially, a 5 mm round sticker was attached to the center of the buccal surface to serve as a site for artificial WSL. The remaining exposed surfaces were coated with clear nail varnish to prevent demineralization. The sticker was removed after a 24 h drying period at room temperature prior to demineralization, thereby exposing the standard enamel window for WSL creation [12].

A sub-surface caries-like lesion was developed by the hydroxyethylcellulose acidified gel technique. The demineralization gel was prepared by adding sodium hydroxide to 0.1 M lactic acid to adjust the solution to pH 4.5. After that, 6% hydroxyethyl cellulose powder (Sigma Aldrich, St. Louis, MO, USA) was added under constant stirring for one hour, subsequently, wallpaper gel consistency was achieved and left for 24 h at 37 °C to complete hydrolysis [13,14]. Forty teeth were assigned identification numbers and allocated into four groups using computer-based randomization (ten samples per group). Groups were allocated based on the exposure duration of demineralization:

Group 1 (G7): 7 days;
Group 2 (G10): 10 days;
Group 3 (G14): 14 days;
Group 4 (G21): 21 days.

The selected durations are based on previous in vitro studies that used varying demineralization durations and refreshment times to generate lesions of variable severity [13,14,15,17,18]. This study includes short, intermediate, and prolonged demineralization to allow for comparisons of lesion depth, surface integrity, and visual and diagnostic readings across progressive stages of WSL formation.

Each tooth is attached to the lid of a 20 mL plastic container filled with 15 mL of demineralization gel and stored in an incubator at 37 °C for the specified period for each group. The demineralizing gel was not changed in G7 and G10. While the G14 gel was refreshed once on day 7, the G21 gel was refreshed twice on days 7 and 14. At the end of each demineralization protocol, the nail varnish was removed with acetone, and the samples were washed with distilled water and stored in a sealed container with moist filter paper to maintain approximately 100% humidity and prevent dehydration until ICDAS II and DIAGNOdent evaluation. Following sectioning, the specimens were kept at the same humidity conditions until SEM preparation and dehydration protocol [14,19].

### 2.4. Visual Assessment (ICDAS II)

ICDAS II was used to evaluate, classify, and confirm the stage of white spot lesions. Prior to the study, examiner calibration for ICDAS II scoring was performed for both the researcher and another well-trained dentist through a theoretical review of ICDAS II criteria and reference images, and a practical examination of representative extracted teeth with varying degrees of demineralization under blind, controlled dry and wet conditions via a standardized visual assessment of WSLs. The scores obtained were compared and discussed until agreement was reached.

During the study and at the end of each demineralization period, the teeth were rinsed with distilled water and examined from the buccal view under controlled light conditions using the same dental operatory LED light source, fixed illumination angle, and distance in wet conditions and after 5 s air-drying [2].

The ICDAS II scoring system is as follows:

- Code (0): No visual change.
- Code (1): First visual change (opacity or discoloration) in enamel is hardly visible on the wet surface, but distinctly visible after air drying.
- Code (2): Distinct visual change (opacity or discoloration) in enamel, visible without air drying.
- Code (3): Enamel breakdown, no dentin visible.
- Code (4): Dentin shadow (not cavitated into dentin).
- Code (5): Distinct cavity with visible dentin.
- Code (6): Extensive distinct cavity with visible dentin [20].

### 2.5. DIAGNOdent Pen Reading

The reading of demineralized WSL was recorded using a laser-induced fluorescence detection device (Diagnodent pen) (KaVo, Biberach, Germany). Before each measurement, the pen is calibrated using a standard ceramic disk (KaVo, Biberach, Germany). According to the manufacturer’s instructions, the fluorescence of the teeth may differ from tooth to tooth and from patient to patient; therefore, the pen is further calibrated and set to a zero reading by placing the pen tip on a clean and clear area of the lingual surface of each tooth. Following this, the probe tip B of the pen is placed in the middle of the lesion created on the buccal surface and then tilted around the measuring area to collect fluorescence from all directions within different demineralization duration groups. This process was repeated three times to guarantee consistency in the readings [21]. Moreover, the measurement process was repeated by another well-trained dentist to assess inter-rater reliability.

### 2.6. Scanning Electron Microscope (SEM) Evaluation of Lesion Depth and Surface Topography

**Figure 2:**
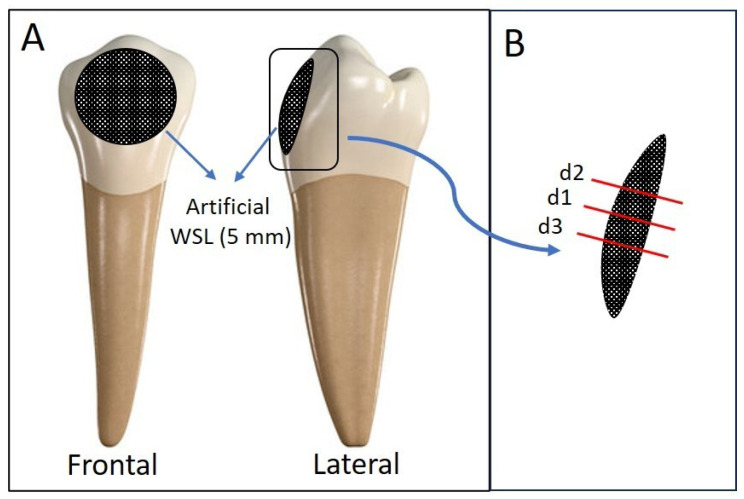
Schematic illustration of WSL depth measurement: (**A**) Frontal and lateral perspectives of a 5 mm diameter artificial WSL at the center of the upper first premolar clinical crown. (**B**) A magnified depiction of the WSL cross-section demonstrating the three measurement points, where d1 is located at the center, and d2 and d3 are each spaced 100 µm apart from the center.

Following the demineralization period, the teeth of each group were sectioned buccolingually at the center of the clinical crown into two halves using a diamond cutting disk mounted on a cutting machine (Marathon, Saeyang Microtech, Republic of Korea) with water cooling. Subsequently, polishing was performed on the cross-section surface of the randomly selected one-half using silicon carbide polishing paper graded at 400, 600, 800, and 1000 grit in sequence under running water coolant to achieve a smooth, shiny finish for accurate microscopic evaluation and lesion depth assessment by minimizing the sectioning artifact, while the buccal surface of the other half was used for qualitative WSL surface texture assessment, with the adjacent undemineralized enamel area serving as the sound surface reference for qualitative SEM comparison to minimize inter-sample and surface variability [22]. Both buccal and cross-sectional surfaces were air-dried at room temperature for 24 h and then gold sputter-coated using a sputter-coating system (CYKY SEM Coating System) at 30 kV and 20 mA. The samples were then examined under SEM (Axia ChemiSEM, Thermo Fisher Scientific, USA) at an accelerating voltage of 30 kV for the operator to measure quantitative lesion depth, scanning blindly to minimize potential measurement bias, and for researcher and a well-trained dentist to assess surface topography qualitatively [23,24]. The depth was measured from the point of greatest buccal curvature at 1500×, with a working distance of 100 µm; three measurements were obtained from the surface to the deepest point of demineralization. The first measurement was taken at the center of the SEM micrograph, and the other two measurements were taken bilaterally at 100 µm from the center. After that, the mean of the measurements was used in the study (Figure 2) [25].

### 2.7. Statistical Analysis

All data from this study were statistically analyzed using SPSS (Statistical Package for the Social Sciences, version 26.0, IBM Corp., Armonk, NY, USA). The normality and homogeneity of data were assessed using the Shapiro–Wilk and Levene’s tests, respectively. For homogeneous data, One-Way Analysis of Variance (ANOVA) with Tukey HSD post hoc was used, while non-homogeneous data were analyzed using Welch and post hoc Games–Howell to test differences between groups. Inter-rater reliability was assessed using the Intraclass Correlation Coefficient (ICC). Spearman’s correlation with two-tailed significance was used to assess the strength of the relationship. Nonparametric variables were tested using Kruskal–Wallis. A *p*-value < 0.05 was considered statistically significant.

## 3. Results

The ICC values for inter-rater reliability DIAGNOdent and lesion depth equal 99.5% and 99.7%, respectively, indicating excellent reliability.

### 3.1. International Caries Detection and Assessment System (ICDAS II)

Table 1 presents the ICDAS II scores for all samples included in this study. In Group 1 (G7), eight samples scored 1, while the remaining two scored 2. In contrast, samples in all other groups consistently exhibited a score 2 WSLs, with no scores of 0, 1, or 3. The Mann–Whitney test showed a statistically significant difference between G7 and the other groups, whereas no significant difference in mean visual scores was observed among the remaining groups.

### 3.2. DIAGNOdent Pen Fluorescence Outcomes

Table 2 shows changes in DIAGNOdent pen readings across different WSL development protocols, with marked differences among the four groups as demineralization periods increased. The lowest fluorescence value was observed in the 7-day protocol (mean: 7.80 ± 2.04), followed by the 10-day protocol (mean: 18.10 ± 3.38). The 14-day and 21-day protocols recorded steep increases in the readings (51.90 ± 11.39 and 82.70 ± 13.11, respectively).

Statistically, the data were normally distributed. However, Levene’s test indicated a violation of the homogeneity of variance assumption; therefore, Welch and Games–Howell post hoc tests were used for multiple comparisons, revealing statistically significant differences among groups (*p* < 0.001), confirming a progressive increase in the DIAGNOdent reading with increased demineralization exposure time.

### 3.3. Scanning Electron Microscope (SEM) Evaluation of Lesion Depth

**Figure 3:**
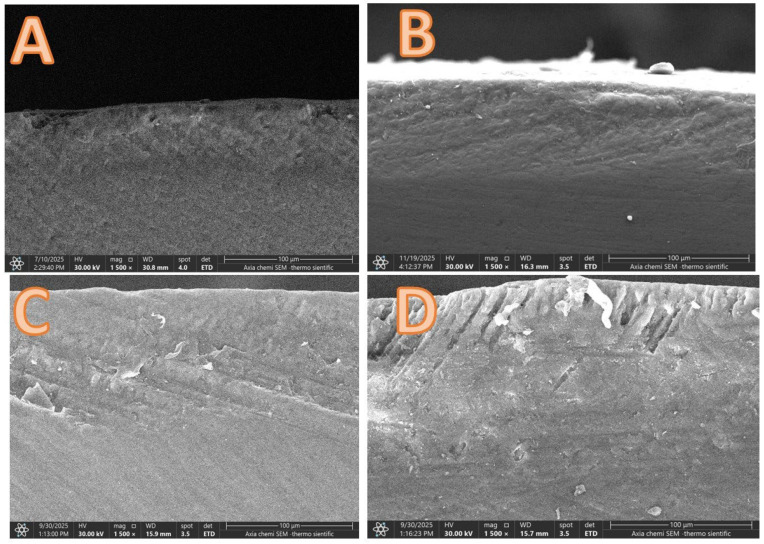
SEM evaluation of enamel lesion depth following various interval protocols: (**A**) a 7-day demineralization period exhibits shallow lesion depth with an intact enamel surface; (**B**) a 10-day demineralization period displays an increased lesion depth with comparable surface to 7-day group; (**C**) a 14-day demineralization period shows more lesion depth with the appearance of surface irregularities; (**D**) a 21-day demineralization period displays extensive micropore development accompanied by the loss of the intact outer surface.

Table 3 and Figure 3 present the SEM analysis of lesion depth across the groups. The shallowest lesion depth mean value (46.59 µm ± 2.72) was observed in the protocol with the shortest exposure time (G7), indicating early subsurface lesion formation. The SEM analysis of the remaining groups, G10, G14, and G21, demonstrates a progressive increase in lesion depth, 69.57 µm ± 4.62, 91.52 µm ± 6.92, and 101.7 ± 9.75, respectively, with increased demineralization time. As homogeneity of variance was confirmed, ANOVA and Tukey HSD were used, confirming a significant effect of increasing exposure to the demineralization gel on producing deeper enamel lesions.

### 3.4. Scanning Electron Microscope Evaluation of the Surface Topography

**Figure 4:**
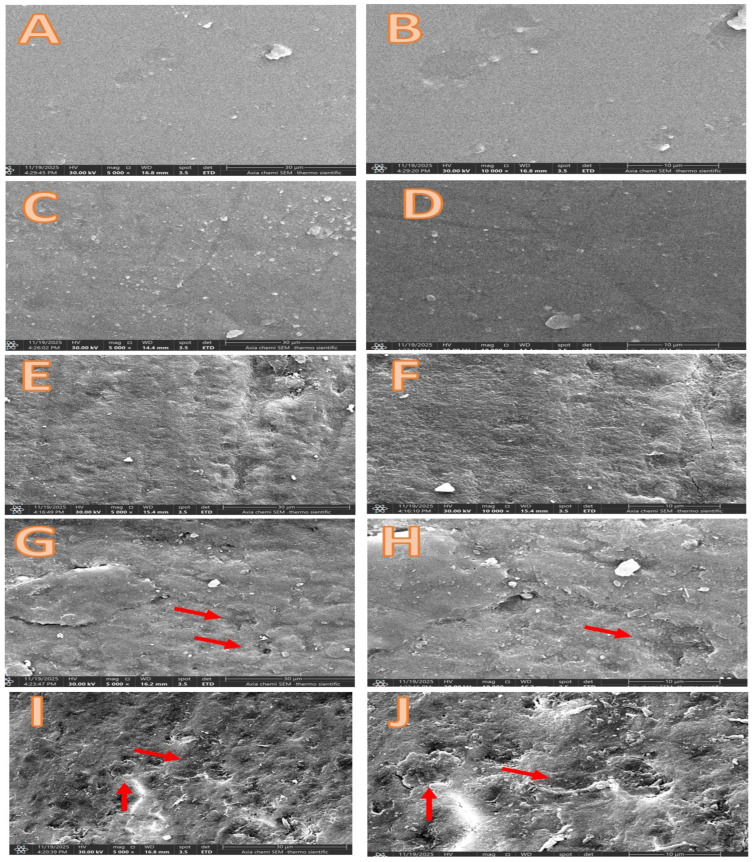
SEM evaluation of surface topography at 5000× (**A**,**C**,**E**,**G**,**I**) and 10,000× (**B**,**D**,**F**,**H**,**J**): (**A**,**B**) control enamel surface; (**C**,**D**) enamel after a 7-day demineralization period displays a smooth surface closely resembling the control group; (**E**,**F**) 10-day demineralization period shows increased surface irregularity; (**G**,**H**) 14-day demineralization period shows enamel surface porosity (red arrows); (**I**,**J**) 21-day demineralization period exhibits loss of surface integrity and abundant micropores (red arrows).

Figure 4 shows SEM micrographs of the control enamel surface and the demineralized enamel surface at each demineralization interval at two magnifications (5000× and 10,000×). The sound enamel (control) surface is clean and smooth (Figure 4A,B). After 7 days of demineralization (G7), the enamel surface exhibits roughness comparable to that of sound enamel (Figure 4C,D). By 10 days (G10), the enamel shows slightly increased surface roughness, with no porosity or cavitation (Figure 4E,F). In contrast, increased demineralization days (G14) result in a marked increase in surface roughness, a widening of the interprismatic space, and enamel prism dissolution, reflecting the formation of shallow pores (red arrows) (Figure 4G,H). On the other hand, a 21-day demineralization period (G21) reveals enamel with more pronounced and extensive porosity, marked prism dissolution, and loss of surface continuity (Figure 4I,J).

### 3.5. DIAGNOdent Pen Score, Lesion Depth, and ICDAS II Correlation

Table 4 presents the correlation between DIAGNOdent readings and both lesion depth and ICDAS II using Spearman’s correlation and two-tailed significance. The results show that DIAGNOdent readings have a very strong, significant, positive correlation with lesion depth (r = 0.90, *p* < 0.001) and a moderate, significant, positive correlation with ICDAS II (r = 0.69, *p* < 0.001). Moreover, lesion depth shows a moderate, significant positive correlation with ICDAS (r = 0.66, *p* < 0.001).

## 4. Discussion

The significance of WSL as a challenging sequela of suboptimal oral hygiene, particularly during fixed orthodontic treatment, has been the focus of numerous experimental and clinical studies. Experimental research holds indispensable importance for the body of knowledge. A robust and meticulous in vitro simulation of natural demineralization processes is crucial for reliable subsequent clinical translation. Enhancing this integrative process ultimately contributes to safer and more efficacious WSL diagnostic and therapeutic strategies in clinical practice. The formation of WSLs on extracted premolars is considered the initial experimental step in evaluating new remineralizing therapies; therefore, they should exhibit surface and subsurface characteristics comparable to those of clinical WSLs. In the present study, acidified hydroxyethyl cellulose was used to create and assess subsurface WSLs at various demineralization durations, with the aim of optimizing these WSLs to closely match the characteristics of clinical post-orthodontic WSLs, thereby producing the deepest WSLs with nearly intact surfaces. The characteristic features of these subsurface WSLs were subsequently evaluated using the ICDAS II system, DIAGNOdent pen, and SEM.

The acidified hydroxyethyl cellulose has been found to be an easy-to-handle gel; the lesions created with this material established consistent depths of demineralization and demonstrate higher rigidity compared to those developed with acetic acid buffer [26]. The latter solution tends to produce deeper, larger, and erosion-like lesions compared to the acidified buffer gel due to its rapid diffusion rate, which prevents mineral reprecipitation and compromises enamel surface integrity. On the other hand, the acidified gel acts as a dental plaque to produce a more controlled demineralization process, and allows for reprecipitation of the dissolved minerals at the surface, producing an intact surface layer that mimics clinical and post-orthodontic lesions [14].

The consistent increase in DIAGNOdent readings with prolonged demineralization periods signifies deeper lesions, increased mineral loss, and accompanying alterations in surface topography. The lowest DD readings observed in Group 1 corresponded to the shortest demineralization duration, aligning with the ICDAS II visual score of 1, which indicates early-stage carious lesions. Notably, the remaining demineralization protocols (Groups 2 to 4) exhibited an ICDAS II score of 2, despite substantial variations in DD readings, thereby emphasizing the limited diagnostic accuracy of the visual scoring system. Conversely, the notable escalation in DIAGNOdent readings underscores the device’s effectiveness in differentiating the successive phases of demineralization. These findings hold particular clinical significance, as both diagnostic methods are routinely employed in clinical research for the identification of WSL; however, the ICDAS II visual scoring system becomes less reliable once it attains a score of 2. These results are corroborated by prior studies indicating that the DIAGNOdent device serves as a valuable supplementary tool for early carious lesion detection beyond the capabilities of visual examination [27].

However, the pronounced increase in DD readings, exceeding 30 degrees in G14 and G21, is inconsistent with the manufacturer-specified interpretation range, indicating that the first two periods produce early, non-cavitated, non-porous enamel changes comparable to clinical WSLS, as confirmed by SEM surface evaluation. At longer demineralization periods, the readings deviated from the manufacturer’s criteria. Although previous study demonstrate an increase in DIAGNOdent readings with longer demineralization periods, these readings do not exceed the manufacturer-recommended range, even with the emergence of surface porosity and erosion-like appearance, which may be due to different demineralization protocols [28]. This deviation may reflect the manufacturer’s thresholds, which are primarily validated for clinical conditions (in vivo). These findings align with those reported in a previous systematic review, highlighting the heterogeneity, moderate specificity and sensitivity, and false-positive readings of the DIAGNOdent device in an in vitro setting [29].

On the contrary, histopathological evaluation is considered the gold standard for assessing in vitro WSL lesion depth [30]. SEM is a widely used histopathological technique in various studies [23,24]. In addition, it has been used to evaluate enamel surface texture and is recognized as a valid and reliable tool for assessing the surface morphology of WSLs [31,32,33,34,35]. Consequently, in this study, SEM readings were used to validate the lesion structure, confirm the most reliable demineralization period, and validate other diagnostic methods.

Herein, the SEM findings confirm progressive mineral loss and increased lesion depth with longer exposure to the demineralization gel, which is consistent with the reported finding that there is strong time-dependent progression [36]. The shallowest lesion was found in the G7, consistent with ICDAS II and DIAGNOdent readings. The SEM depth values in this study align with those reported in a previous study under similar demineralization conditions [19]. The most profound lesion, exceeding 100 µm, was identified in the G21 group, indicative of a time-dependent escalation in lesion depth. Nevertheless, the rate of depth advancement progressively diminished with an extended demineralization period. Lesion depth approximately doubled when exposure duration was increased from 7 to 14 days, rising from 46.59 µm to 91.52 µm. However, extending the exposure to 21 days resulted in only a marginal increase of approximately 20%, reaching 101.7 µm, in comparison to the 7-day baseline. This phenomenon may be attributable more to greater surface mineral loss than to increased lesion depth and could reflect the emergence of surface porosity and disintegration, as observed in the SEM evaluation of surface topography in both the G14 and G21 groups. This may serve as a plausible explanation for the deviation in DIAGNOdent readings, which exceed the threshold specified by the manufacturer and are corroborated by existing literature.

Although a strong positive correlation was observed between the depth of the SEM lesions and DIAGNOdent measurements, which corroborates findings from a prior systematic review and meta-analysis supporting the association of increased fluorescence with deeper lesions [7]. It remains plausible that this correlation is primarily attributable to the increased surface porosity and disintegration that occur with prolonged demineralization, rather than to lesion depth itself. Consequently, this may account for the heterogeneity observed in DIAGNOdent’s performance across various in vitro studies [7]. Alternatively, the increase in DIAGNOdent readings shows inconsistent correlation with higher ICDAS II scores, as visual assessments did not surpass an ICDAS II score of 2. These findings are further supported by a randomized controlled trial that found a significant discrepancy between DIAGNOdent results and visual evaluations of the same treated lesions, reinforcing the conclusion that fluorescence methods have greater sensitivity to subsurface alterations than visual methods [37]. Nevertheless, it is recommended to use fluorescence as an adjunct to visual examination rather than as a replacement, thereby supporting diagnostic sensitivity while preserving the clinician’s reliance on visual assessment skills [38].

The null hypothesis was rejected due to the presence of statistically significant differences in WSL characteristics (surface and depth) across experimental groups with increasing demineralization durations, as verified by ICDAS II, DIAGNOdent, and SEM assessments. Furthermore, a significant correlation was identified among the ICDAS II score, SEM-measured WSL depth, and DIAGNOdent fluorescence values. As with any in vitro experimental model, this study has the limitation that it does not fully reflect the complex oral environment, where factors such as bacterial biofilms, pH fluctuations, dietary challenges, and oral prophylaxis mechanisms may affect lesion formation. Another limitation is the heterogeneity in the DIAGNOdent readings. Moreover, inter-examiner reliability statistics (e.g., Cohen’s kappa) for ICDAS II scores were not calculated.

## 5. Conclusions

The in vitro WSL created with acidified hydroxyethyl cellulose and a 10-day demineralization protocol demonstrated favorable lesion characteristics relevant to the orthodontic WSL under controlled laboratory conditions, with its surface remaining nearly intact and clear evidence of subsurface demineralization. The developed G10 WSL maintained reproducibility and consistency in accordance with DIAGNOdent criteria. Consequently, these lesions have the potential to enhance the process of WSL diagnosis and the evaluation of new intensive remineralization therapies prior to clinical implementation.

The deterioration of surface integrity and the emergence of enamel porosity in G14 and G21 WSLs tend to amplify DIAGNOdent readings, potentially accounting for the strong correlation observed between DIAGNOdent measurements and lesion depth. On the other hand, ICDAS II demonstrates lesser sensitivity to depth variations in comparison to DIAGNOdent. Further studies are recommended to evaluate the optimized white spot lesion with other demineralizing solutions, such as acetic buffer solution, and to use additional diagnostic methods such as micro-CT, TMR, and Raman spectroscopy for more comprehensive characterization.

## Figures and Tables

**Table 1:** Descriptive and inferential statistics of the International Caries Detection and Assessment System (ICDAS II) after different demineralization periods.

Groups	N	ICDAS II Scores				Statistics: Kruskal–Wallis	Statistics: Mann–Whitney
		0	1	2	3		
G7	10	0	8	2	0	H = 29.250 d*f* = 3 *p* < 0.001	A *
G10	10	0	0	10	0		B *
G14	10	0	0	10	0		B *
G21	10	0	0	10	0		B *

(*) Dissimilar letters indicate significant differences among groups at the 0.05 level. The significance values were adjusted by the Bonferroni correction for multiple tests.

**Table 2:** Descriptive and inferential statistics of DIAGNOdent pen reading after different demineralization periods.

Groups	N	Min	Max	Mean	SD	Statistics: Welch	Statistics: Games–Howell Post Hoc
G7	10	4	11	7.80	2.04	H = 150.620 d*f* = 3 *p* < 0.001	A *
G10	10	13	22	18.10	3.38		B *
G14	10	31	69	51.90	11.39		C *
G21	10	66	99	82.70	13.11		D *

(*) Dissimilar letters indicate significant differences among groups at the 0.05 level.

**Table 3:** Descriptive and inferential statistics of the lesion depth evaluated by SEM after different demineralization periods.

Groups	N	Min	Max	Mean	SD	Statistics: ANOVA	Statistics: Tukey HSD Post Hoc
G7	10	43.80	50.61	46.59	2.72	F = 139.608 d*f* = 3 *p* < 0.001	A *
G10	10	63.15	77.83	69.57	4.62		B *
G14	10	79.64	101.85	91.52	6.92		C *
G21	10	81.96	116.27	101.7	9.75		D *

(*) Dissimilar letters indicate significant differences among groups at the 0.05 level. All lesion depth data in micrometers (µm).

**Table 4:** Spearman’s correlation among the DIAGNOdent pen score, SEM lesion depth, and ICDAS II correlation.

Spearman’s Correlation		SEM Lesion Depth	ICDAS II
DIAGNOdent scores		0.904	0.693
	Sig. (two-tailed)	0.000	0.000
	N	40	40
SEM lesion depth			0.666
	Sig. (two-tailed)		0.000
	N		40

## Data Availability

The data that support the findings of this study are available from the corresponding author upon request.
